# ASSOCIATIONS BETWEEN NEIGHBORHOOD CHARACTERISTICS AND SITTING PATTERNS IN OLDER ADULTS

**DOI:** 10.1093/geroni/igz038.075

**Published:** 2019-11-08

**Authors:** Mikael Anne Greenwood-Hickman, Rod L Walker, KatieRose Richmire, Andrea LaCroix, Eric B Larson, Paul K Crane, Dori E Rosenberg

**Affiliations:** 1 Kaiser Permanente Washington Health Research Institute, Seattle, Washington, United States; 2 University of California San Diego, La Jolla, California, United States; 3 Department of Medicine, University of Washington, Seattle, Washington, United States

## Abstract

Neighborhood characteristics are associated with self-reported sedentary behavior (SB) in older adults. However, self-report measures are not able to accurately assess total sitting time nor detailed patterns of SB. This analysis explores the relationship between device-based SB variables from activPAL and neighborhood characteristics (demographics) in the ACT cohort. Neighborhood characteristics were assessed with the modified Physical Activity Neighborhood Environment Scale (PANES; scored 1.0-4.0, higher score, higher walkability). Data were analyzed using linear regression models adjusted for demographic factors. Higher PANES score was associated with higher daily steps (+1180 daily steps/point on PANES, p<0.001) and sit-to-stand transitions (+2.7 daily transitions/point on PANES, p=0.004). Confirming other studies, neighborhood walkability promotes physical activity. A novel finding was that sitting interruptions, which can only be assessed with devices, were also associated with higher neighborhood walkability, while total sitting time was not.

